# Exploring Feature Selection and Classification Techniques to Improve the Performance of an Electroencephalography-Based Motor Imagery Brain–Computer Interface System

**DOI:** 10.3390/s24154989

**Published:** 2024-08-01

**Authors:** Md. Humaun Kabir, Nadim Ibne Akhtar, Nishat Tasnim, Abu Saleh Musa Miah, Hyoun-Sup Lee, Si-Woong Jang, Jungpil Shin

**Affiliations:** 1 Department of Computer Science and Engineering, Bangamata Sheikh Fojilatunnesa Mujib Science & Technology University, Jamalpur 2012, Bangladesh; humaun@bsfmstu.ac.bd (M.H.K.); s19111128@bsfmstu.ac.bd (N.I.A.); s19211106@bsfmstu.ac.bd (N.T.); 2 School of Computer Science and Engineering, The University of Aizu, Aizuwakamatsu, Fukushima 965-8580, Japan; 3 Department of Applied Software Engineering, Dongeui University, Busanjin-Gu, Busan 47340, Republic of Korea; 4 Department of Computer Engineering, Dongeui University, Busan 47340, Republic of Korea

**Keywords:** Feature Selection, Brain-computer Interface (BCI), Electroencephalography (EEG), Motor Imagery (MI), Relief-F, Linear Discriminant Analysis (LDA), Machine Learning (ML)

## Abstract

The accuracy of classifying motor imagery (MI) activities is a significant challenge when using brain–computer interfaces (BCIs). BCIs allow people with motor impairments to control external devices directly with their brains using electroencephalogram (EEG) patterns that translate brain activity into control signals. Many researchers have been working to develop MI-based BCI recognition systems using various time-frequency feature extraction and classification approaches. However, the existing systems still face challenges in achieving satisfactory performance due to large amount of non-discriminative and ineffective features. To get around these problems, we suggested a multiband decomposition-based feature extraction and classification method that works well, along with a strong feature selection method for MI tasks. Our method starts by splitting the preprocessed EEG signal into four sub-bands. In each sub-band, we then used a common spatial pattern (CSP) technique to pull out narrowband-oriented useful features, which gives us a high-dimensional feature vector. Subsequently, we utilized an effective feature selection method, Relief-F, which reduces the dimensionality of the final features. Finally, incorporating advanced classification techniques, we classified the final reduced feature vector. To evaluate the proposed model, we used the three different EEG-based MI benchmark datasets, and our proposed model achieved better performance accuracy than existing systems. Our model’s strong points include its ability to effectively reduce feature dimensionality and improve classification accuracy through advanced feature extraction and selection methods.

## 1. Introduction

Despite its infancy, brain–computer interface (BCI) technology has the potential to revolutionize the IT industry by enabling users to control computers directly with their brains, resulting in user-friendly systems [1]. Brain–computer interfaces (BCIs) have become very important in neuro-engineering and neuroscience because they help people recover from strokes by using neuroplasticity to improve their communication and make it easier for disabled people to communicate by analyzing emotions, detecting events, and keeping an eye on their sleep [2]. This technology allows individuals with paralysis to control devices, motorized wheelchairs, or prosthetic limbs using their thoughts. Electrodes for invasive BCIs require surgical brain implantation. These offer better signals, but come with their own risks. Non-invasive BCIs, on the other hand, can pick up and record brain impulses without surgery using methods such as an EEG, functional near-infrared spectroscopy (fNIRS), and magnetoencephalogram (MEG), but the signals they pick up are often less clear [3]. The BCI interprets sensory input from peripheral nerves, triggering voluntary or automated actions. It measures central nervous system activity and converts it into an output for computer use. The BCI aids both humans and animals by facilitating functions such as reasoning, learning, and language comprehension. This system can also control breathing successfully. BCI technology has emerged as a pivotal element in human-computer interaction, enabling users to control devices and interact with their environment using brain activity. This technology utilizes EEG to capture and analyze neurophysiological data, allowing real-time system adaptation based on the user’s mental state. It is particularly beneficial for individuals with motor disabilities, facilitating communication and operation of prosthetic devices through motor imagery tasks [1,4,5,6,7]. Detecting real-world human activity during the MI job is the primary objective of the BCI-based applications, which aim to convert human thought processes into equivalent digital commands that various types of devices can operate. Researchers have been attempting to extract useful characteristics while looking for a suitable machine learning or deep learning-based algorithm for classification. To classify MI tasks, we applied the CSP feature extraction technique, one of the most popular feature extraction algorithms, to the EEG data [2,8,9,10,11]. In medical signal processing, especially in healthcare, CSP is used to change EEG data from time to space. The goal is to find spatial weights that separate data groups by using linear algebra and multivariate statistics [12]. The study uses CSP feature extraction algorithms to extract EEG signal characteristics and build high-dimensional feature fusion. The CSP takes characteristics to break down multivariate signals and projects EEG multichannel data into a low-dimensional subspace of space. This approach maximizes variance and minimizes variation within one class, improving class discrimination. Originally, the CSP algorithm was used to identify aberrant EEGs and efficiently classify movement-related EEGs in [13]. The problem with MI-based BCI (MI-BCI) is that the brain signals that turn the mental image of movement into instructions are very different from person to person. The CSP algorithm is a good way to tell the difference between two types of EEG readings in the MI-BCI system [14,15]. To apply the CSP algorithm effectively, we need to provide several factors: the band-pass filter at the frequency of EEG measurements, the time interval during which we obtain EEG measurements about the stimuli, and the subset of CSP filters we will employ. Usually, we utilize two or three subsets of CSP filters, with the time segment starting one second after the cue and the frequency spectrum of 7–30 Hz as generic settings. Subject-specific parameters, however, might increase the effectiveness [16]. To overcome the challenges, we proposed an efficient and comprehensive framework for motor imagery task classification that integrates multiple advanced techniques for feature extraction, selection, and classification. This comprehensive approach positions our methodology as a powerful tool for EEG signal classification, particularly in distinguishing motor imagery activities. The major advantages of the proposed framework are (a) enhanced feature extraction, (b) robust feature selection, (c) classifier diversity, (d) computational efficiency, (e) generalizability, (f) proven performance on benchmark datasets, and (g) real-world applicability. Below, we outline our contributions to the proposed work.

- **Enhanced feature extraction:** We split the EEG signal into four bands and then used CSP to obtain useful features from each narrow band. Multiband Decomposition allows us a more detailed analysis of frequency-specific information. This multiband approach captures both spatial and frequency domain features more comprehensively than single-band methods. We utilized the CSP technique within each sub-band to extract narrowband-oriented effective features. This results in a high-dimensional feature vector that is more discriminative and capable of improving classification performance.
- **Robust feature selection:** High-dimensional feature space can reduce the effectiveness of the machine learning algorithm and increase its computational complexity. To solve the issues, we employed an efficient feature selection approach, namely Relief-F. This effectively gives us a low-dimensional effective feature space leading to improved performance classification algorithms and reduced computational complexity.
- **Classifier diversity:** Finally, we fed the reduced feature vector into the classification algorithm to generate the probabilistic maps, aiming to leverage their respective strengths, enhancing the robustness and accuracy of the classification results. We implemented a diverse set of advanced classification algorithms (SVM, LDA and MLP) to process the reduced feature vector and tested the performance of Relief-F algorithm.
- **Efficiency and generalizability:** The preprocessing and feature selection steps significantly reduce the computational complexity, making the proposed method suitable for real-time applications. The extensive experiments demonstrated that the proposed model outperformed with three different benchmark EEG datasets in terms of performance accuracy, AUROC, F1-score, and computational time, thereby demonstrating its strength.

## 2. Literature Review

In the past few decades, numerous studies have examined on BCIs for classifying MI tasks. Different research groups have used brain signals from the motor cortex area, using different ways to look at EEG data for BCI applications across channels and looking into how experimental paradigms work physiologically. Pfurtscheller et al. applied Linear Discriminant Analysis (LDA) combined with Adaptive Autoregressive (AAR) for classifying left- and right-hand motor imagery EEG signals. LDA serves as a statistical method for dimensionality reduction and classification. AAR likely helps to capture temporal dependencies in the EEG data [17]. Researchers commonly use CSP as an optimal spatial filter. CSP extracts a weighted score for each electrode based on its significance in discriminating between different classes (e.g., left vs. right-hand motor imagery). By identifying important electrodes, CSP enhances classification accuracy [18].

There are some drawbacks to the broad frequency range. The methods mentioned primarily focus on a wider range of frequencies in EEG signals. However, a narrower frequency band may be more effective for specific tasks. Researchers often divide the broader EEG signal into subbands (e.g., mu, beta, alpha, and gamma rhythms) to capture task-specific information [19,20]. Ramos et al. demonstrated that the most effective approach for classifying motor imagery tasks involves combining the Genetic Algorithm with the LDA classifier. Electroencephalography (EEG) is a prominent, non-invasive method for capturing brain signals. Developing an EEG-based MI-BCI encompasses preprocessing, feature extraction, selection, and classification stages. The study looks at and compares six feature selection methods (CFS, Consistency, Relief-F, mRmR, C4.5, and Genetic Algorithm) that were used on EEG data for the MI task classification. This shows how important feature selection is for getting the best classification results. The evaluation incorporates five widely used classifiers: PNN, RBF, SVM, LDA, and KNN [21].

The paper by Thomas et al. [6] talks about the Discriminative Filter Bank Common Spatial Pattern (DFBCSP) algorithm as a way to make EEG-based BCIs better at classifying motor imagery tasks. However, one potential drawback of the method is that it may require further validation and testing on a larger and more diverse population of subjects to ensure its generalizability and robustness across different individuals and conditions. Also, even though the DFBCSP algorithm seems better at lowering error rates than other methods, more research is needed to see how well it works in real-time or online BCI applications, and how useful and efficient it is in changing environments. Wang et al. made a way to use Relief-F and enhanced Lasso together to obtain wavelet packet entropy features and topological details of the brain function network from raw MI EEG data in their study. They performed signal denoising, channel filtering, wavelet soft thresholding, and one-to-one multi-class score CSP methods. They then extracted relative wavelet packet entropy and topological features using the multi-core Lasso and Relief-F methods. The method was applied to two public EEG datasets, the BCI Competition III dataset IIIA and the BCI Competition IV dataset IIA, for classification purposes. The results showed that the strategy preserved EEG data and reduced computing complexity. This method could be useful in rehabilitation and MI-BCI applications. However, the paper also discusses the potential drawbacks of motor imagery classification methods, such as dimensionality issues, redundancy, scalability concerns, feature selection challenges, and computational complexity. Large dimensions may impact screening results, while fusion of features may introduce redundancy and increase computational demands. Scalability may reduce classification accuracy, and feature selection may vary depending on the dataset and task [19,22]. In [23], the Filter Bank Common Spatial Pattern (FBCSP) algorithm was developed by Ang et al. It is a machine-learning method for processing motor imagery EEG signals in BCIs. The FBCSP algorithm selects discriminative CSP features from a bank of filters and spatial filters and then uses a feature selection algorithm to classify the selected features. This method outperforms existing methods, such as Sub-band Common Spatial Pattern (SBCSP) and CSP with manually selected operational frequency bands, in terms of classification accuracy. However, FBCSP faces challenges such as computational complexity for managing high-dimensional feature vectors and requiring high-quality input data.

According to Kabir et al., the Multi-Subspace Randomization and Collaboration-Based Unsupervised Feature Selection (SRCFS) method, along with the classifier LDA worked best for sorting MI tasks on the two public BCI Competition III datasets, IVA and IIIB. This study looks into BCI by using different feature selection methods and both traditional and machine learning-based classifiers on EEG signals. The main goal is to improve the classification of MI tasks. The proposed method’s average classification accuracy was 90.05%. The paper suggests an effective way to select and classify features for MI-based EEG signals in the BCI paradigm, but it still has a lot of problems, such as being hard to compute, having a lot of noise and artifacts, and working with high-dimensional feature vectors [2]. Molla et al. used a CSP feature extraction method and then a nearest-neighbor-based discriminative feature selection method to pick the potential features and leave out the garbled features to improve MI classification using a multichannel EEG signal. Some problems with this method are that it only works for a certain amount of time for EEG trials, it does not pick the best features, it is not specific enough for NCFS methods, it depends on labeling training data, and it cannot be used in real-time situations [24].

In [25], Venkatachalam et al. proposed a Hybrid-Kernel Extreme Learning Machine (KELM) method based on Principal Component Analysis (PCA) and Fisher’s Linear Discriminant (FLD) for the MI BCI classification of EEG data. The major limitations of this paper are limited generalizability and interpretability, and sensitivity to noise. Tiwari et al. suggested using an automatic EEG channel selection for multiclass MI classification to simplify the processing of numerous channels. The study combines the objective Firefly Algorithm (FA) and Fisher information to create a hybrid channel ranking process. They extract spatial-temporal features from preprocessed brain signals using the Regularized Common Spatial Pattern with Aggregation (RCSPA) approach. Weighted scores for each channel are calculated near a potential solution using objective FA and input variables (Spectral Entropy and Lyapunov exponent). A novel Channel Set Relevance Index (CSRI) ranks channels based on their weighted scores and Fisher information. The Regularized Support Vector Machine (RSVM) classifier utilizes the RCSPA properties of highly ranked channels to differentiate between various MI tasks. The method is validated using three publicly available BCI competition datasets with different channel counts, showing improved classification accuracy (83.97% on dataset 1, 80.85% on dataset 2, and 84.19% on dataset 3) compared to baseline approaches while using fewer channels [26].

We proposed a method for subject-specific frequency range band-pass filtering for EEG measurements to address the problems listed in this research, enabling better classification using motor imagery. We incorporated the SVM, LDA, and MLP classifiers along with the Relief-F, ILFS, Inf-FS, FSV, and SD feature selection methods. The LDA and Relief-F-based MI task classification systems work better than those for three different MI EEG datasets. Relief-F is a binary classification algorithm that handles numerical or discrete features. By determining feature scores, which can serve as feature weights, Relief-F ranks and selects the best features. The algorithm’s feature scoring is based on finding feature value discrepancies between closest neighbor instances. We have improved performance on noisy issues, multi-class problems, and incomplete data. The main goals are to enhance performance, scalability, adaptability to different data types, and efficiency.

## 3. Dataset Description

To evaluate our model, we used three benchmark datasets for EEG-based MI-BCI classification. These are BCI Competition III Dataset IVA, Dataset IIIB, and Dataset IIIA, which are described in Section 3.1, Section 3.2, and Section 3.3, respectively. We have extensively analyzed the generalizability property using these two datasets.

### 3.1. BCI Competition III Dataset IVA

The BCI competition III dataset IVA is a valuable resource for researchers, practitioners, and industrial personnel working on BCIs. Fraunhofer initially provided the dataset, sourced from the Intelligent Data Analysis Group and the Neurophysics Group at the Benjamin Franklin Campus of Charité-University Medicine Berlin [27]. The BCI Competition III dataset IVA focuses on motor imagery tasks, specifically the imagination of left-hand and right-hand movements. In this context, the classification problem is to distinguish between these two types of motor imagery tasks. This is a binary classification problem, where the two classes are class 1: imagining left-hand movement and class 2: imagining right-hand movement. The experimental setup involved five healthy subjects who completed four activities with binary classifications being considered, as depicted in Table 1. This table summarizes the training and testing trials for the BCI Competition III dataset IVA. During recording, the electrodes on the subject’s scalp were set up using the global 10–20 system, and visual cues indicated which subject should perform. The dataset consisted of continuous signals from 118 EEG channels with 0.05–200 Hz frequency range and markers indicating the time points of 280 cues for each subject. The dataset includes training and test trials for each subject, aiming to develop effective BCI algorithms using limited training data. Participants recorded motor imagery tasks in a calm state, taking into account their movements. Trials were timed between 1.25 and 2.25 s, digitized at 1000 Hz, filtered, and downsampled at 100 Hz for 0.5–3 s in each cue.

### 3.2. BCI Competition III Dataset IIIB

The BCI competition III dataset IIIB is another widely used and fascinating dataset that contributes to the advancement of MI-EEG-BCI systems. Here, cued motor imagery using three to four sessions from three individuals and online feedback (non-stationary classifier) with two classes (two bipolar channels in the EEG). The dataset is composed of recordings from three subjects, S4, X11, and O3, each with varying trials. The dataset is divided into test and training datasets to maximize performance for unknown test labels. The three electrodes used to collect this dataset were applied to the subject’s scalp by the international 10–20 system. A 7 s the recorded signal is the basis of a trial signal. A variety of trials were gathered from the various subjects. For example, 320 trials were conducted on the O3 subjects, while 1080 trials were obtained from S4 and X11, respectively. The recorded signal was sampled at a frequency of 125 Hz and subsequently filtered using a notch filter with a bandwidth of 0.5 to 30 Hz [28]. Due to the use of virtual reality (VR) in the experiment for the O3 subject, we have excluded this subject from the performance evaluation.

### 3.3. BCI Competition III Dataset IIIA

BCI Competition III Dataset IIIA is a motor imagery multi-class dataset with four classes (left hand, right hand, foot, and tongue), three subjects (K3b, K6b, and L1b), 64 EEG channels with a 1–50 Hz frequency range, 250 Hz sampling rate, and 60 trials per class. In this experiment, we developed a binary classifier to distinguish two types of motor imagery tasks: imagining left-hand movement (class 1) and imagining right-hand movement (class 2).

## 4. Proposed Approaches

**Figure 1:**
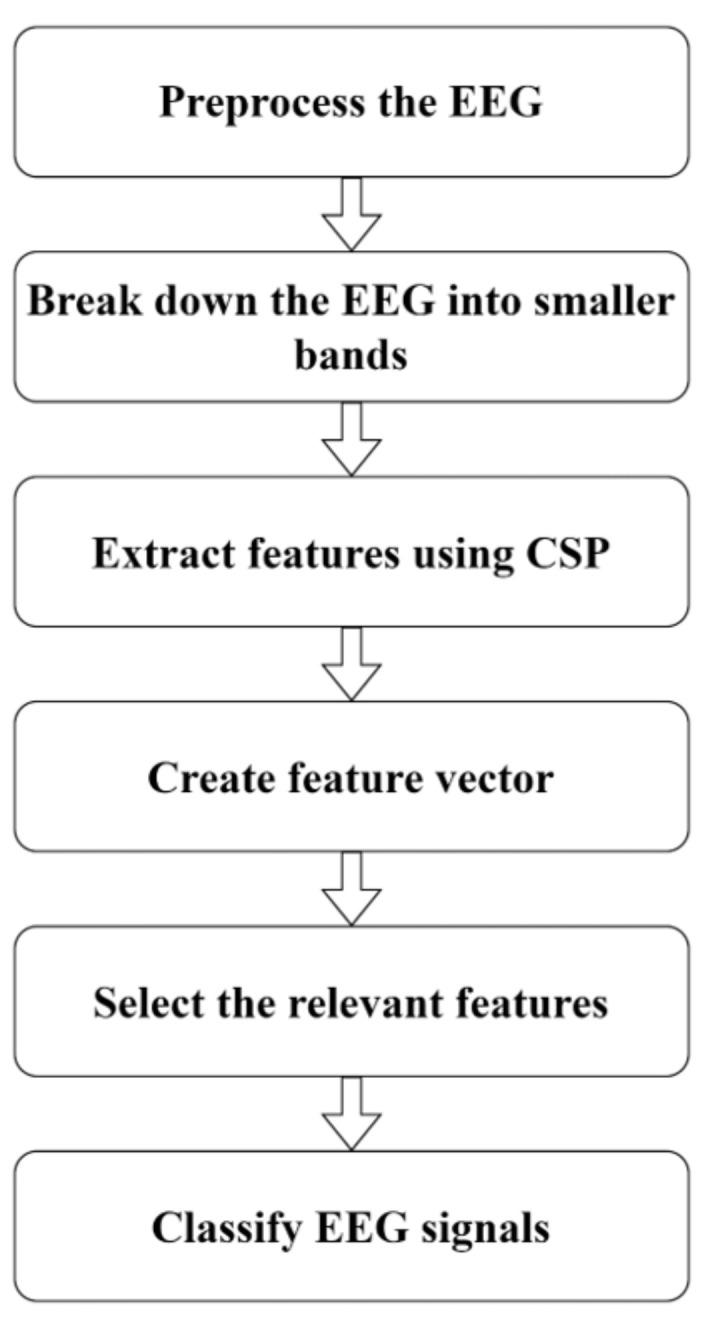
Workflow of proposed method.

To complete our work, we have employed the following procedures which are illustrated in Figure 1. By properly following them, we have implemented our research work. The steps are as follows.

**Step 1—preprocessing and division of signals:** The raw EEG signals are preprocessed to remove noise and artifacts, resulting in clean multichannel EEG signals. These preprocessed signals are then divided into multiple narrowband signals to facilitate the extraction of more effective features.

**Step 2—filter bank analysis:** Each EEG signal trial is broken down into smaller frequency bands using filter bank analysis. This step allows us to capture detailed information across different frequency ranges, enhancing the granularity of the feature extraction process.

**Step 3—feature extraction:** The CSP method is employed to extract spatial information from each sub-band. CSP is known for its effectiveness in identifying patterns that maximize the variance between different classes, thereby improving the discriminative power of the features.

**Step 4—formation of feature vector:** The spatial features extracted from each subband are combined to form a comprehensive feature vector. This step integrates information across all subbands, ensuring that the feature vector encapsulates the EEG signals’ spatial and frequency domain characteristics.

**Step 5—feature selection:** Feature selection algorithms are applied to the combined feature vector to enhance the performance and reduce computational complexity. We use multiple algorithms, including Relief-F, Inf-FS, ILFS, SD, and FSV, to identify the most potential and discriminative features. This selection process ensures that only the most relevant features are retained, resulting in a reduced and more robust feature vector.

**Step 6—classification:** The final reduced feature vector is fed into classifiers to distinguish between different motor imagery (MI) activities. We utilize a combination of Support Vector Machine (SVM), Linear Discriminant Analysis (LDA), and Multilayer Perceptron (MLP) classifiers. Each classifier brings unique strengths: SVM for its strong generalization capabilities, LDA for its efficiency in linear separable data, and MLP for its ability to model complex non-linear relationships.

### 4.1. Preprocessing

In this study, we have applied several pre-processing schemes to the raw EEG input data, including filtering to remove undesirable signals like noises and artifacts and selecting the channel for further processing. EEG data processing requires the filtering of EEG waves, as the interpretation of brain activity relies on MI EEG signals. Raw EEG consists of various kinds of noises and artifacts, such as eye blinking, sudden sounds, muscle movements, body movements, environmental noises, and so on. Common filtering techniques include band-pass filtering, wavelet transform, and notch filters. The BCI Competition III Dataset IVA, Dataset IIIB, and Dataset IIIA were recorded with the frequency ranges 0.05–200 Hz, 0.5 to 30 Hz, and 1–50 Hz, respectively. In the preprocessing step, we cleaned and filtered the following three datasets and used a bandwidth of 8 to 30 Hz. Following this, the EEG signal was segmented into four equivalent narrowband signals: Mu-band (8–13 Hz), low-beta (13–22 Hz), high-beta (22–30 Hz), and full-band (8–30 Hz) for further analysis, because the majority of brain activity associated with MI tasks occurs in the 8–30 Hz frequency range. Mu-band (8–13 Hz), a category of the alpha band with the same frequency range, is specifically associated with the sensory-motor cortex [2,29,30]. We created the high-dimensionality feature vector by combining the retrieved CSP-based features from each subband [31,32].

### 4.2. Feature Extraction

In this research, we apply the CSP to extract EEG signal characteristics and build high-dimensional feature fusion. Medical signal processing typically uses CSP to transform EEG data from time to space, with the goal of identifying spatial weights that differentiate two or more EEG data groups. By putting EEG multi-channel data into a low-dimensional space subspace and giving each line a channel weight, the CSP method makes the range of two-class signal matrices bigger. This method enhances class discrimination by reducing intra-class variance and enhancing inter-class variation, as noted by [13]. We employed the CSP algorithm as a spatial filter to make high-variance features between the right-hand and right-foot classes, leading to peak variances between those classes. Let $E_{c1}^{i^{\prime }}$ and $E_{c2}^{i^{\prime }}$ be an EEG signal of the $i^{th}$ trial, and $c_{1}$ and $c_{2}$ represent the class 1 and class 2. The projection matrix $W_{\mathrm{CSP}}$ is determined by initially computing the normalized spatial covariance matrix for each class, as shown in Equations (1) and (2).

(1) $$C_{L}=\frac{E_{c1}E_{c1}^{\prime }}{\mathrm{trace}(E_{c1}E_{c1}^{\prime })}$$

(2) $$C_{R}=\frac{E_{c2}E_{c2}^{\prime }}{\mathrm{trace}(E_{c2}E_{c2}^{\prime })}$$

where $E^{\prime }$ denotes the transpose of *E*, the averaged normalized covariances $C_{L}$ and $C_{R}$ are calculated by averaging all segments within each class. The total composite spatial covariance is then obtained from the sum of $C_{L}$ and $C_{R}$, as $C_{c}=C_{L}+C_{R}$. This covariance matrix is factorized into its eigenvalues and eigenvectors as $C_{c}=U_{c}\lambda_{c}U_{c}^{\prime }$. The eigenvector matrix and diagonal eigenvalue matrix, in this case, are arranged in descending order and are represented by the symbols $U_{c}$ and $\lambda_{c}$, respectively. We can compute the whitening transformation using the following Equation (3):

(3) $$P=\sqrt{\lambda_{c}^{-1}U_{c}}$$

where *P* stands for the whitening transformation. The common spatial pattern can be calculated from the covariance matrix of the two classes according to Equation (4):

(4) $$W_{CSP}=P^{\prime }B=\left[w_{1},w_{2},...,w_{\left(ch-1\right)}w_{ch}\right]\in R^{\left(ch\times ch\right)}$$

In this equation, the variable ch is the channel, and B is an orthonormal matrix. A matrix $W_{CSP}=\left[w_{1},w_{2},...,w_{2m}\right]\in R^{\left(2\times k\right)}$ consists of spatial filters that represent the k biggest and smallest eigenvalues obtained by solving Equation (5). The final feature can be expressed as $f=[f_{1},f_{2},\ldots ,f_{2k}]$

(5) $$f_{j}=log(var\left({W^{\prime }}_{CSP}E_{i}\right),j=1,2,...,2k$$

In this equation, the variance is represented by var(.), and log transformation is used for normalizing the elements of $f_{j}$.

### 4.3. Feature Selection

Due to its complexity and numerous electrodes, the EEG signal often contains irrelevant data. Eliminating unnecessary features is crucial for the implementation of EEG-based MI-BCI systems. Recent research focuses on improving classification performance using existing features. However, the large combined multiband feature dimension can increase computational complexity and reduce performance. Certain features can degrade the performance of traditional classifiers or machine learning algorithms. Feature selection strategies are divided into filter and wrapper approaches [33], and can be performed online or offline. For feature sets of moderate size, the execution time is not a major concern due to the offline nature of the feature selection process. However, data mining and classification applications have recently used over a thousand features. In this case, a feature selection method must consider its computing time. This study used newly developed and effective feature selection approaches for MI classification and examined five feature selection methods: Relief-F, ILFS, Inf-FS, FSV, and SD. In our analysis, the Relief-F feature selection algorithm demonstrated superior performance compared to other methods due to its ability to handle noise, multi-class problems, and incomplete data efficiently. The following subsections provide a concise overview of each feature selection technique.

#### 4.3.1. Relief-F Feature Selection

Relief-F is a filter-based feature selection technique introduced in 1992 by Kira and Rendell for binary classification tasks. It works with both numerical and discrete features. The algorithm determines feature relevance by considering how much it differentiates between classes and is independent of other features. We achieve this evaluation by analyzing the differences in feature values between pairs of closest neighbor instances. Relief-F can improve classification performance when there are noise issues, multi-class problems, or incomplete data. The main goals of new versions and extensions are to enhance performance, make it more scalable, adapt to different data types, and improve efficiency [34]. In the Relief-F feature selection method, if $x_{r}$ and $x_{q}$ features belong to the same class, the predictor weight update formula can be represented as Equation (6):

(6) $$W_{ji}=W_{ji-1}-\frac{\Delta_{j}\left(x_{r},x_{q}\right)}{m\cdot d_{rq}}$$

If $x_{r}$ and $x_{q}$ are in different classes, it can be determined using Equation (7):

(7) $$W_{ji}=W_{ji-1}+\frac{p_{y_{q}}\left(1-p_{y_{r}}\right)\cdot \Delta_{j}\left(x_{r},x_{q}\right)}{m\cdot d_{rq}}$$

where $W_{ji}$ is the weight of predictor at the $i_{th}$ iteration step, $\Delta_{j}\left(x_{r},x_{q}\right)$ is the absolute difference between the $j_{th}$ feature of $x_{r}$ and $x_{q}$ normalized by the range of the $j_{th}$ feature, and $d_{rq}$ is a scaled distance function between $x_{r}$ and $x_{q}$. The $x_{r}$ refers to one instance characterized by its features and $x_{q}$ refers to another instance, which is also characterized by its features. The scaled distance function can be defined as Equation (8):

(8) $${\tilde{d}}_{rq}=e^{-{\left(\mathrm{rank}\left(r,q\right)/\sigma \right)}^{2}}$$

where rank(r, q) is the position of the $q_{th}$ observation among the nearest neighbors of the $r_{th}$ observation sorted by distance and σ is a parameter that affects the scaling. Total distance $d_{rq}$ can be calculated by the following Equation (9):

(9) $$d_{rq}=\sum_{l=1}^{k}{\tilde{d}}_{rl}$$

#### 4.3.2. Infinite Feature Selection (Inf-FS)

Infinite Feature Selection (Inf-FS) is a graph-based technique that evaluates a feature’s importance using the convergence characteristics of a power series of matrices. Features are represented as nodes, feature relationships as edges, feature subsets as paths, and infinite path exploration. The frequency of each feature’s appearance in high-scoring paths, strongly associated with good classification performance, determines its importance. We prioritize features based on their scores, where higher scores signify greater significance. When choosing features from an EEG, each extracted feature in the Inf-FS graph is treated as a node, and the right metrics are used to judge them, such as correlation, coherence between electrodes or channels, or mutual information. The feature importance scores provide insight into the relative contributions of various features. Inf-FS effectively minimizes redundant features by considering their overall impact, is versatile, and requires thorough assessment and validation before use in machine learning works [35].  In the case of pairwise feature analysis, assuming a set of feature distributions F={f(1),…,f(n)} and a sample x∈R representing a distribution *f*, then we can construct an undirected fully connected graph G=(V,E), where the parameter *V* denotes the collection of vertices for each feature distribution, and *E* represents weighted edges indicating pairwise relations among feature distributions. The adjacency matrix A of G encodes pairwise energy terms as in Equation (10):

(10) $$a_{ij}=\lambda {\%}_{ij}+\left(1-\lambda \right)\rho_{ij}$$

where λ is a loading coefficient in the range [0,1], ${\%}_{ij}=\max\left(\sigma \left(i\right),\sigma \left(j\right)\right)$ measures maximal feature dispersion, and $\rho_{ij}=1-\mathrm{Spearman}\left(f\left(i\right),f\left(j\right)\right)$ measures Spearman’s rank correlation coefficient. For a path $P=\{v_{0}=i,v_{1},\ldots ,v_{l-1},v_{l}=j\}$ of length *l* between vertices *i* and *j*, the energy of feature subsets $E_{P}$ is determined using the following Equation (11):

(11) $$E_{P}=\prod_{k=0}^{l-1}a_{v_{k},v_{k+1}}$$

The energy of every path between *i* and *j* of length *l* can be calculated utilizing Equation (12):

(12) $$R_{l}\left(i,j\right)=\sum_{P\in P_{i,j}^{l}}E_{P}$$

The energy associated with feature f(i) at path length *l* can be calculated using Equation (13):

(13) $$s_{l}\left(i\right)=\sum_{j\in V}R_{l}\left(i,j\right)$$

For infinite feature sets, the definition of the geometric series is $S=\sum_{l=1}^{\infty }A^{l}$, and the energy of feature f(i) considering infinite paths can be determined by the following Equation (14):

(14) $$s\left(i\right)=\sum_{l=1}^{\infty }s_{l}\left(i\right)={\left[Se\right]}_{i}$$

Regularization using the generating function can be expressed as Equation (15):

(15) $$\hat{s}\left(i\right)=\sum_{l=1}^{\infty }r^{l}s_{l}\left(i\right)=\sum_{l=1}^{\infty }r^{l}\sum_{j\in V}R_{l}\left(i,j\right)$$

Computation using the convergence property of geometric series can be formulated as Equations (16) and (17):

(16) $$\hat{S}={\left(I-rA\right)}^{-1}-I$$

(17) $$\hat{s}\left(i\right)={\left[\hat{S}e\right]}_{i}$$

The Inf-FS method follows the several steps shown in Algorithm 1:
**Algorithm 1** Inf-FS method1:Build the graph and compute adjacency matrix A.2:Let paths tend to infinity.3:Choose a regularization factor *r*.4:Compute $\hat{S}={\left(I-rA\right)}^{-1}-I$.5:Compute $\hat{s}=\left[\hat{S}e\right]$.6:Return $\hat{s}$ as the energy scores for each feature.

This approach allows for analyzing pairwise relations among features, computing feature subset energies, and determining feature importance by considering infinite paths. The algorithm provides a systematic way to compute energy scores for feature selection.

#### 4.3.3. Infinite Latent Feature Selection (ILFS)

We rank features in EEG channels according to the importance of their neighbors using a probabilistic technique known as Infinite Latent Feature Selection (ILFS). The algorithm uses the geometric power series of a matrix and a simple generating function for the path. It aims to check the validity and reliability of study findings, analyze and empirically clarify the importance of important qualities ranked highly by the ILFS, and evaluate the resilience of the suggested technique. ILFS works by modeling latent variables, building the probabilistic model, and acquiring knowledge of model parameters using inference algorithms. The posterior probability distribution indicates the likelihood of each latent variable influencing the observed data, with features with higher probabilities being more relevant. ILFS enhances interpretability by offering insights into the latent variables that underlie feature associations, and by assessing the relevance of each feature to the overall task goal through the use of latent variables. To apply MI EEG, you have to find features, make latent variables, find the model parameters, and rank features by how well they can tell the difference between latent states [36]. The steps working behind the ILFS method is properly mentioned in the Algorithm 2:
**Algorithm 2** ILFS Method1:The function LearningGraphWeights (X,Y,TT,verbose) computes the adjacency matrix A, representing relationships among pairs of features, by learning weights from input data X and labels Y.2:The code establishes a regularization factor, r, derived from the maximum eigenvalue of the adjacency matrix A, ensuring convergence of the infinite series. A, ensuring convergence of the infinite series3:Calculate the matrix S using Gelfand’s formula, representing the convergence of the geometric series of matrices4:Compute the sum of each row of matrix S to obtain the energy scores for each feature. These scores represent the relevance or importance of each feature5:Rank the features based on their energy scores in descending order

We can implement the algorithm using the following mathematical equations. Adjacency Matrix *A* representing relationships among features can be obtained by using the following formula at Equation (18):

(18) A=LearningGraphWeights(X,Y,TT,verbose)

Regularization factor *r* for this can be gained by Equation (19):

(19) $$r=\frac{\mathrm{factor}}{\rho }$$

where ρ is the maximum eigenvalue of *A*. Equation (20) determines the convergence of the geometric series of matrices *S*:

(20) $$S={\left(I-rA\right)}^{-1}-I$$

where *I* is an Identity matrix. To estimate the energy scores, WEIGHT, we used Equation (21):

(21) $$WEIGHT=\sum_{i}S_{i}$$

where $S_{i}$ represents the *i*-th row of matrix *S*. Finally, the ranking features RANKED is calculated using Equation (22):

(22) $$RANKED=\mathrm{sort}(WEIGHT^{\prime },descend^{\prime })$$

These equations summarize the key steps of the ILFS algorithm, from learning graph weights to ranking features based on their energy scores.

#### 4.3.4. Feature Selective Validation (FSV)

The Feature Selective Validation (FSV) method is a widely used validation tool in electromagnetic measurements and models, particularly in electrical systems. It uses Monte Carlo analysis to move uncertainty from experimental data to FSV quantities, which makes sure that the results are reliable because they are not linear. FSV optimizes feature selection for MI EEG by combining the learning automaton and firefly algorithm, increasing classification accuracy by removing unnecessary elements. It also efficiently removes redundant features, improving classification accuracy. The technique’s viability in real-world BCI systems is confirmed by real-time studies [37]. The FSV technique involves several steps, including the preprocessing of data, calculation of the Amplitude Difference Measure (ADM), the Feature Difference Measure (FDM), and ultimately, the Global Difference Measure (GDM). In the preprocessing phase, we utilized the following steps:

- Decompose the original data vector *x* into three portions: DC, Lo, and Hi. The baseline or low-frequency portion of the data may be referred to as DC. Following transformations and filtering procedures, the low-frequency data component is represented by Lo. The high-frequency data component that results from filtering procedures is called Hi.
- Apply Fourier transform (DFT) to obtain frequency domain components.
- Determine the index $I_{b}$, where the low-frequency portion amounts to 40% of the total.
- Implement linear tapering across $N_{b}$ samples for vector separation across $I_{b}$.

Now, the calculation of FSV indexes can be performed using the following formulae. First, we have to calculate ADM and FDM indexes. The ADM calculates the absolute differences between intensity values at each point and compares them to determine the amplitude or intensity difference between two datasets. FDM is a statistical method that quantifies differences in data features or shapes, comparing first derivatives to assess their variation. So, Equation (23) of FDM can be written as follows:

(23) $$\mathrm{FDM}=\frac{\sum_{x_{\min}}^{x_{\max}}\left|I_{{\mathrm{set}1}^{\prime }}\left(x\right)\right|}{\sum_{x_{\min}}^{x_{\max}}\left|I_{{\mathrm{set}1}^{\prime }}\left(x\right)-cI_{{\mathrm{set}2}^{\prime }}\left(x\right)\right|}$$

In this equation, $I_{{\mathrm{set}1}^{\prime }}\left(x\right)$ and $I_{{\mathrm{set}2}^{\prime }}\left(x\right)$ represent the first derivatives of the datasets concerning *x*, where $x_{\min}$ and $x_{\max}$ define the range of *x* values over which the comparison is performed, and *c* is the ratio of the average intensities of the datasets. The GDM, a single metric that evaluates the overall similarity or dissimilarity between datasets, combines information from both ADM and FDM. To compute the GDM index, we combine the ADM and FDM indexes using the following Equations (24) and (25):

(24) $$GDM_{i}=\sqrt{{\left(ADM_{i}\right)}^{2}+{\left(FDM_{i}\right)}^{2}}$$

(25) $$GDM=\sqrt{\sum_{i=0}^{N-1}GDM_{i}}$$

In these equations, $GDM_{i}$ represents the GDM index for the *i*-th dataset, ADMi represents the ADM index, and FDMi represents the FDM index.

Based on this Algorithm 3, the overall FSV method can be presented by Equation (26):

(26) $$\mathrm{FSV}=\mathrm{sort}\left(-\left|w\right|\right)$$

where FSV represents the Feature Similarity Validation score, and *w* represents the weights assigned to each feature by the FSV algorithm. This equation captures the essence of the FSV algorithm, where features are ranked based on the absolute magnitude of their weights assigned during the feature selection process.
**Algorithm 3** FSV Method1:Initialize parameters and variables.2:Iterate through the main loop until convergence or a maximum number of iterations3:Update weights w using linear optimization.4:Sort features based on the absolute magnitude of weights to obtain the feature ranking.

#### 4.3.5. Statistical Dependency (SD)

The Statistical Dependency feature selection technique is also called the statistical significance-based feature selection method. Statistical significance-based feature selection methods are a filter method in machine learning used to reduce dimensionality by identifying features with a significant relationship with the target variable. The method starts with a null hypothesis $\left(H_{0}\right)$ and applies statistical tests to each feature–target pair, such as chi-square for categorical data or correlation for numerical data. A *p*-value, which indicates the likelihood of witnessing the data under the null hypothesis, is produced by the test. A significance level α is chosen, and features with *p*-values below α are considered statistically significant and likely relevant to the target variable. However, features with *p*-values greater α than may be excluded. Statistical significance indicates a stable relationship, not necessarily a strong one, and may overlook weaker relationships or interactions with other features [38]. Statistical Dependency (SD) between features and labels measure whether feature values depend on associated class labels or co-occur by chance. Features are quantized into quantization scale (QS) levels. An adaptive quantization scale ensures that each bin contains roughly equal samples across the dataset. The statistical dependency between the discrete feature values (*y*) and class labels (*z*) is evaluated using the following Equation (27):

(27) $$SD=\frac{\sum_{y\in Y}\sum_{z\in Z}p\left(y,z\right)}{p(y)p(z)}$$

In this equation, *Y* represents the quantized feature values, p(y,z) denotes the joint occurrence frequency of a feature value and a class label, p(y) and p(z) represent the probabilities of feature values and class labels, respectively.

A larger SD indicates a higher dependency between feature values and class labels. The minimum value of 1 indicates complete independence. SD is preferred in certain cases due to its sensitivity to highly informative quantization levels.

### 4.4. Classification Using LDA, SVM and MLP

At present, machine learning and neural network-based approaches are crucial and common in biomedical research for identifying signals like EEGs, which are essential for understanding cognitive functioning and detecting brain diseases. The initial stage involves identifying prominent features in unprocessed EEG signals, which serve as identifiers for classification. Biomedical research, where accurate classification of EEG signals is crucial for cognitive comprehension and diagnosis, uses a methodology similar to this one. In this study, we used three popular and widely adopted machine learning-based classification algorithms, namely LDA, SVM, and MLP, to classify left-hand and right-hand human motor imagery EEG signals. The goal is to determine which yields the best results.

Linear discriminant analysis (LDA), normal discriminant analysis (NDA), or discriminant function analysis is a way for statistics and machine learning to find a linear combination of features that tells two or more groups of objects or events apart. LDA finds the best way to separate classes by projecting the data onto a lower-dimensional space and finding a set of linear discriminants that make the difference between the differences within and between classes as high as possible. It assumes equal covariance matrices and a Gaussian distribution, as well as the assumption that the data are linearly separable. To create a new axis, LDA uses two criteria: maximizing the distance between the means of two classes and minimizing the variation within each class. The computation process involves calculating the between-class variance and the within-class variance and projecting the data into a lower-dimensional space to maximize the between-class variance and minimize the within-class variance [39,40].

Support Vector Machines (SVMs) are powerful supervised learning algorithms for classification and regression tasks. Introduced by Vapnik, SVMs are particularly effective in solving problems with complex decision boundaries. Unlike traditional linear classifiers, SVMs aim to find the optimal hyperplane that separates data points of different classes while maximizing the margin, which is the distance between the hyperplane and the nearest data points of each class. One key concept in SVMs is the use of nonlinear maps to transform the input data into a higher-dimensional space, where it becomes linearly separable. This transformation enables SVMs to handle nonlinear relationships between features. The hyperplane determined by SVMs is defined by a subset of training data points called support vectors, which lie closest to the decision boundary. SVMs solve a dual optimization problem involving Lagrange multipliers, where the objective is to maximize the margin while minimizing classification errors. By solving this optimization problem, SVMs effectively identify the support vectors and determine the optimal hyperplane. In practice, SVMs offer flexibility in choosing different kernel functions, such as the radial basis function (RBF) kernel, which allows SVMs to capture complex relationships in the data. The RBF kernel measures the similarity between data points in the transformed feature space, enabling SVMs to handle non-linearity and achieve high classification accuracy. Overall, SVMs are versatile and widely used in various applications, including image classification, text categorization, and bioinformatics, due to their ability to handle high-dimensional data and nonlinear relationships effectively [41].

A multilayer perceptron (MLP) neural network is a supervised learning algorithm that computes output and was initially defined by Frank Rosenblatt. MLP moves forward with every node connected, using the backpropagation algorithm to improve training model accuracy. MLP has three primary layers: the input layer, the hidden layer, and the output layer. The input layer receives weighted inputs from earlier layers, applies an activation function, and outputs a value. The hidden layer learns complex associations between features and class labels, while the output layer contains one neuron for each class. MLPs are flexible, adaptable, and interpretable, making them useful for EEG classification of various architectures and hyperparameters. The hidden layers used in our experiment are ten in size [42].

### 4.5. Experimental Setting

K-fold cross-validation is a method for evaluating prediction models where a dataset of K folds is divided into training, testing, and validation sets. This approach helps to evaluate, select, and tune the hyperparameters to improve the effectiveness of each model. It protects from overfitting and creates a generalized model. The data set is divided by K and trained and tested in K times using k-fold cross-validation each time. We employed a 5-fold cross-validation technique, randomly dividing the dataset into five equal subsets for cross-validation. The final performance score was obtained by averaging the accuracy over five runs. The formula used to compute the accuracy in percentage (%) is as follows:

$$\mathrm{Accuracy}=\frac{T_{p}+T_{n}}{T_{p}+T_{n}+F_{p}+F_{n}}\times 100$$

where $T_{p}$ = True Positive, $T_{n}$ = True Negative, $F_{p}$ = False Positive, and $F_{n}$ = False Negative. The accuracy results from the several trials primarily demonstrate how successful the suggested strategy is. Then, the performance of the Relief-F feature selection method was compared with the outcomes of some distinct feature selection techniques, namely FSV, Inf-FS, ILFS, and SD. We used SVM and MLP in addition to LDA to critically assess the performance of the classifiers. To validate the reliability and efficiency of the proposed method, we calculated various statistical performance measures on two datasets. These measures include computational time, Area Under the Receiver Operating Characteristic curve (AUROC), and F1 score for each subject. The F1 score is the harmonic mean of precision and recall, providing a balanced assessment of a model’s performance. It symmetrically represents both precision and recall in one metric. The formula for calculating the F1 score is

$$F1=\frac{2\cdot T_{P}}{2\cdot T_{P}+F_{N}+F_{P}}$$

The terms AUC and ROC stand for “area under the curve” and “receiver operating characteristics curve,” respectively. You can also refer to it as the Area Under the Receiver Operating Characteristic Curve, a critical metric for assessing a classification model’s performance.

## 5. Experimental Result

We utilized three benchmark publicly accessible EEG-based MI task datasets to assess the performance of our proposed model. We extracted the precise information from each trial of the dataset by decomposing it into four narrowband signals. A high-dimensional feature vector is generated by extracting features from each narrow band and combining them using the CSP technique. The spatial information is extracted by running each frequency band through the CSP, and as a result, the CSP characteristics are obtained from each of the four bands in the dataset. The BCI competition III dataset IVA and BCI competition III dataset IIIB are combined to form 32 (4 × 8) dimensional feature vectors and 8 (4 × 2) dimensional feature vectors for each trial. Next, the feature selection approaches based on Relief-F, Inf-FS, ILFS, FSV, and SD are applied to the high-dimensional feature space to choose the discriminative characteristics of EEG data. Then, two machine learning-based classifiers, SVM and LDA, and another neural network-based classifier, MLP, are trained independently using the acquired features. After that, the performance of the classifiers is evaluated and validated using test data. The EEG data are extracted for each individual after 2.5 s of trial. All feature selection methods ranked the features based on a variety of parameters. For classification, we selected the number of features that scored the highest.

### 5.1. Performance Result with BCI Competition III Dataset IVA

**Figure 2:**
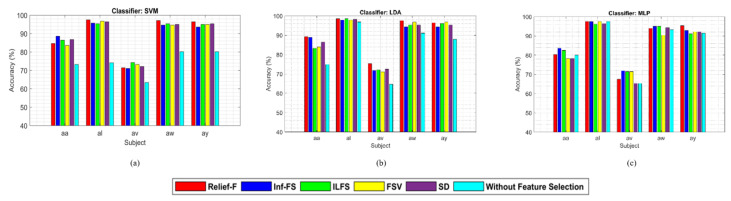
Performance comparison of Relief-F, Inf-FS, ILFS, FSV, SD feature selection methods and without feature selection for MI tasks classification on the BCI competition III dataset IVA with (**a**) SVM, (**b**) LDA, and (**c**) MLP classifiers, respectively. ‘Without feature selection’ means that we did not use any feature selection method; we used the classifiers only to classify the extracted features.

Figure 2 demonstrates the performance comparison of different feature selection methods where SVM, LDA, and MLP classifiers are used, respectively.

**Figure 3:**
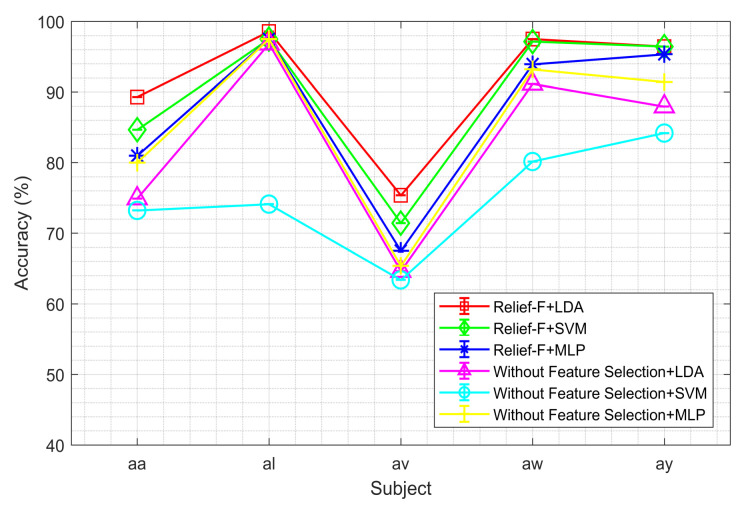
Performance comparison among the LDA, SVM, and MLP classifiers for MI tasks classification using the Relief-F and without feature selection approach demonstrating the accuracy of different subjects on the BCI competition III dataset IVA. ‘Without feature selection’ means that we did not use any feature selection method; we used the classifiers only to classify the extracted features.

These figures demonstrate that the Relief-F feature selection method generally outperforms others with various classifiers, though its performance with the MLP classifier is not consistently superior. However, the performance of the MLP classifier is comparable to other feature selection methods. Figure 3 demonstrates that Relief-F, using the LDA classifier, outperforms the other methods for the BCI competition III dataset IVA. The outcome further confirms the potential advantages of the feature selection method for improving classification performance. Compared to other approaches that employ feature selection techniques, the mean accuracy across all participants significantly decreases in the absence of feature selection. The approach that does not use feature selection reduces classifier performance by including unnecessary characteristics.

**Figure 4:**
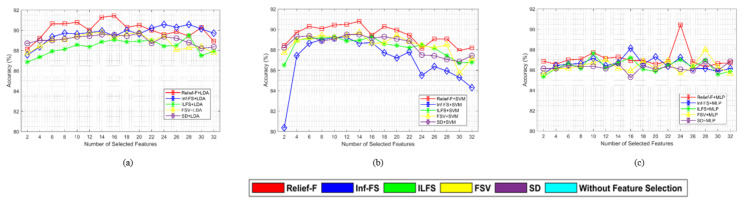
Performance comparison of the MI tasks classification using Relief-F, Inf-Fs, ILFS, FSV, and SD feature selection methods with (**a**) LDA, (**b**) SVM, and (**c**) MLP classifiers for different numbers of selected features. The three subplots represent the accuracies of the BCI competition III dataset IVA for different numbers of features (50% to 100%) selected by the feature selection algorithms.

Figure 4 compares the accuracy of the suggested methods with various feature selection and classifier combinations, where the number of selected features is the determining factor. The three different subplots (a, b, and c) demonstrate that using thirty-two (32) carefully selected features from the BCI competition III dataset IVA achieves the most accurate object classification performance. Here, we obtained the best accuracy by selecting fewer features, which is very helpful to make the system robust and faster. This is true for five different feature selection schemes with three different classifiers. These figures show that the Relief-F feature selection method performed best with three different classifiers (SVM, LDA and MLP) with a really small number of features.

### 5.2. Performance Result with BCI Competition III Dataset IIIB

**Figure 5:**
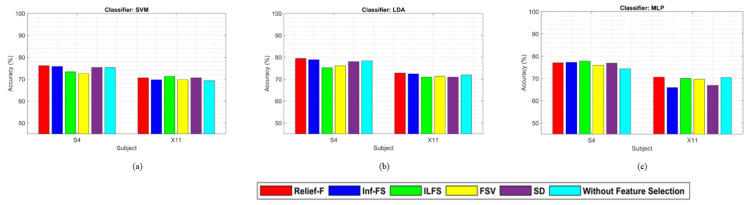
Performance comparison among (**a**) SVM, (**b**) LDA, and (**c**) MLP classifiers for MI tasks classification with the Relief-F, Inf-FS, ILFS, FSV, SD feature selection approaches and without feature selection using the BCI competition III dataset IIIB. ‘Without feature selection’ means that we did not use any feature selection method, we used the classifiers only to classify the extracted features.

In the second dataset, Figure 5 shows the performance of various feature selection methods. Meanwhile, the Relief-F feature selection method generally performs well. It is important to note that the accuracy without using any feature selection method is near to other feature selection methods in the case of BCI Competition III dataset IIIB, because there are only two channels. Also, the number of features is very low. We used this second dataset to confirm the performance with different datasets and the significant generalizability property of the suggested technique. The proposed approach has demonstrated robustness in both datasets for MI task recognition and MI-EEG BCI system implementation.

**Figure 6:**
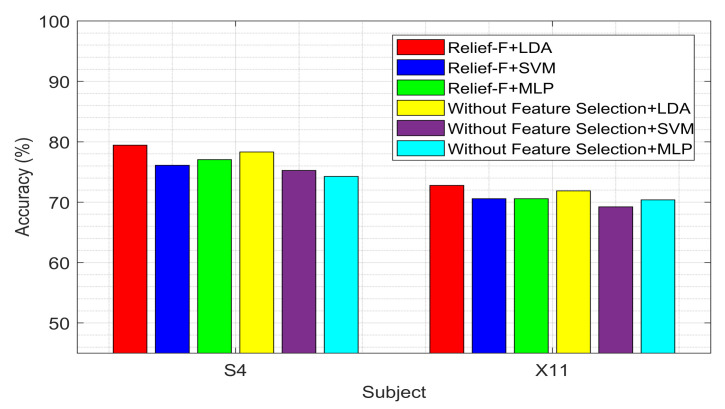
Performance comparison of SVM, LDA, and MLP classifiers for MI tasks classification utilizing the Relief-F feature selection approach and without feature selection. The accuracy of several subjects for the BCI competition III dataset IIIB is shown in the figure. ‘Without feature selection’ means that we did not use any feature selection method, we used the classifiers only to classify the extracted features.

In Figure 6, we can see that our proposed method, Relief-F, outperforms other feature selection methods with the LDA classifier. It also performed better when we tried to classify features without using any feature selection method.

**Figure 7:**
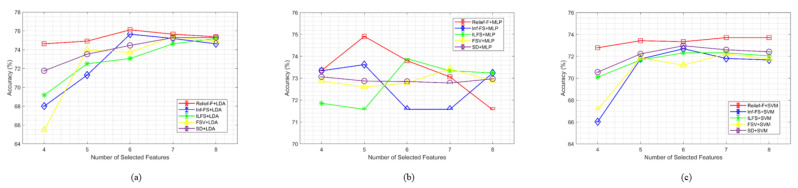
Performance comparison of motor imagery classification utilizing LDA, MLP, and SVM classifiers for varying numbers of selected features, and feature selection techniques such as Relief-F, Inf-FS, ILFS, FSV, and SD. The accuracy of the BCI competition III dataset IIIB is shown in sub-plots (**a**–**c**) for varying feature counts (50% to 100%) chosen by the feature selection technique, which additionally makes use of LDA, MLP, and SVM classifiers.

Figure 7 illustrates a comparison of the accuracy of the suggested method with various feature selection and classifier combinations, where the number of selected features is the determining factor. Figure 7’s subplots (a, b, and c) demonstrate that using five (5) carefully selected features from dataset BCI competition III dataset IIIB achieves the most accurate object classification. The demonstration extends to all three classifiers and all five feature selection techniques. Based on these figures, it is also clear that the Relief-F method outperforms the others in most cases using very low number features.

The non-stationarity of BCI Competition III Dataset IIIB and its restricted information from only two channels are the reasons for its lesser accuracy (76.111%) as compared to BCI Competition III Dataset IVA (91.432%). On the other hand, BCI Competition III Dataset IVA’s 118 channels and consistent statistical characteristics allow for better feature extraction and classification, leading to increased accuracy. We mainly used the second dataset to ensure the generalizability and robustness of our proposed EEG-MI-BCI system. We have also computed numerous statistical performance evaluation metrics to confirm the effectiveness of our proposed MI-EEG-BCI system. We have computed performance metrics such as the area under the ROC, F1 score, and computational time for various subjects on the BCI competition III dataset IVA and dataset IIIB. In this context, we quantify the computational time in seconds (s), which represents the time required for the classifier to train and classify a single fold using a five-fold cross-validation methodology.

### 5.3. State-of-the-Art Comparison with Previous Methods

Table 2 illustrates a comprehensive performance comparison of our proposed approach with ten recently developed algorithms published in previous years. Table 2 clearly shows that our proposed method’s average classification accuracy is 91.432%, and after comparison, we found that the proposed method Relief-F with LDA outperforms the accuracy of the state-of-the-art works. We also looked at how well our suggested method worked against two other methods: the optimal channel and frequency band-based CSP feature selection method by Ming et al. [43] and the Logistic S-shaped Binary Jaya Optimization Algorithm (LS-BJOA) by Tiwari et al. [26]. Once again, our suggested Relief-F feature selection method with the LDA classifier has done a better job than these papers in terms of accuracy.

Ang et al. [23] proposed a CSP-based FBCSP method, where they considered a frequency range of 4–40 Hz, which was split into multiple sub-bands with 4 Hz increments (e.g., 4–8 Hz, 8–12 Hz, 12–16 Hz, 16–20 Hz, and so on up to 36–40 Hz). They then independently applied Common Spatial Patterns (CSP) to each sub-band to extract features. After feature extraction, the Mutual Information-based Best Individual Feature (MIBIF) selection method was used to identify the most effective features. Finally, these selected features were utilized in machine learning algorithms, including Bayesian Theorem, Fisher’s Linear Discriminant (FLD), and Support Vector Machine (SVM), for classification purposes. They reported 90.03% accuracy with the BCI competition III dataset, where our proposed method split the full band signal into four subbands: Mu-band (8–13 Hz), low-beta (13–22 Hz), high-beta (22–30 Hz), and full-band (8–30 Hz). Then, we extracted CSP-based features from the applied feature selection Relief-F method to select the potential features and, using the machine learning method, our model achieved 91.43% accuracy with the same dataset.

By analyzing the accuracy and the above figures, we can conclude that Relief-F with LDA achieves the highest accuracy among all classifiers. This is achieved by keeping the number of selected features low, reducing memory consumption and complexity for both datasets. Even for the BCI competition III dataset IVA, we have carefully selected 32 features. Still, we can obtain the highest accuracy with fewer features using the Relief-F feature selection method, and the accuracy is 91.432%. For the BCI competition III dataset IIIB, by using five carefully selected features, we can obtain the highest accuracy with fewer features using the Relief-F feature selection method, and the accuracy is 76.111%. Since the Relief-F method produces the best result with the LDA classifier for the maximum feature values for the BCI competition III dataset IVA and the BCI competition III dataset IIIB, we can say Relief-F with the LDA is the best for these MI classification tasks. Other performance parameters, including AUROC, F1 Score, and Computational Time for the BCI competition III dataset IVA (Table 3, Table 4 and Table 5) and BCI competition III dataset IIIB (Table 6, Table 7 and Table 8), clearly demonstrate the strength and efficiency of the Relief-F with LDA-based MI task classification system.

**Figure 8:**
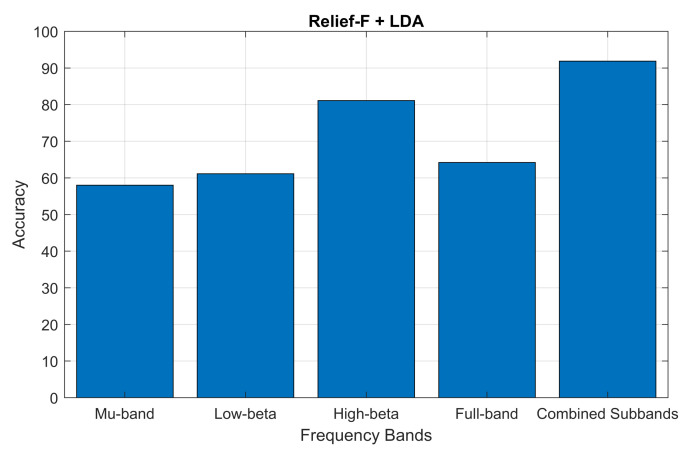
Accuracy performance of individual sub-bands (Mu-band, low-beta, high-beta, and full-band) and combined subbands using BCI competition III dataset IIIA for the subject K3b, respectively.

In addition, we also tested our proposed Relief-F and LDA-based system using a large dataset called BCI Competition III Dataset IIIA for further confirmation of the robustness, effectiveness and generalizability. In the case of the third dataset, we have achieved an accuracy of 91.89 for the first subject K3b, where Wang et al. reported an accuracy of 87.84 in a recently published article [53] for the subject K3b using the Relief-F technique. Moreover, we assessed the accuracy performance of individual sub-bands and combined bands using BCI competition III dataset IIIA for the subject K3b. In the case of the combined band, we considered the different four-frequency bands. Figure 8 below demonstrates the sub-band-wise performance accuracy. In our methodology, we chose specific subbands (Mu-band, low-beta, high-beta, and full-band) based on their known significance in BCI research. We conducted comparative experiments to evaluate the performance of each subband and other combinations [23]. In the experiment results, we observed that our performance accuracy of the four combined subband features is better than the performance of individual subband features.

## 6. Discussion

The study focuses on identifying the optimal combination of feature selection and classification methods for classifying left and right-hand motor imagery (MI) based on EEG signals. We used two publicly available benchmark datasets: BCI Competition III Dataset IVA and BCI Competition III Dataset IIIB. The primary research question was to determine whether the proposed method could improve the classification accuracy of MI tasks using EEG signals. Our results indicate that the Relief-F feature selection method combined with the LDA classifier achieves superior performance compared to other combinations. Specifically, Figure 2 and Figure 3 show subject-wise accuracy, while Figure 4 displays feature-wise accuracy of various feature selection methods (Relief-F, Inf-FS, ILFS, FSV, and SD) with different classifiers (SVM, LDA, and MLP) using BCI Competition III Dataset IVA. The Relief-F with LDA combination achieved the highest accuracy, AUROC values, F1 scores, and the lowest computational time, demonstrating its effectiveness in extracting relevant features and improving classification performance. The superior performance of the Relief-F algorithm can be attributed to its robustness and efficiency in handling noisy and irrelevant data. Relief-F evaluates the importance of features based on their ability to distinguish between neighboring instances, capturing dependencies between features and identifying those most relevant to the target variable. This method’s noise robustness, consideration of feature interactions, and computational efficiency make it well-suited for EEG signal analysis, where data can be noisy and complex. Our approach achieved an accuracy of 91.43% on the BCI Competition III Dataset IVA, compared to the 90.03% accuracy reported by Ang et al. [23] using their FBCSP method with MIBIF. This improvement underscores the effectiveness of our selected frequency bands and feature selection method in capturing discriminative information for MI classification. The findings from Table 3, Table 4 and Table 5 indicate that the Relief-F and LDA combination consistently achieved the highest performance metrics for several subjects in Dataset IVA. Similarly, Figure 5 and Figure 6 show that Relief-F with LDA provides higher accuracy for subjects S4 and X11 in Dataset IIIB. Higher AUROC values, F1 scores, and lower computational times for these subjects, as shown in Table 6, Table 7 and Table 8, respectively, further confirm the effectiveness of this combination. These results suggest that the Relief-F method is highly effective for MI classification tasks, significantly advancing the development of more efficient BCI systems. We assessed the accuracy performance of individual sub-bands and combined bands using BCI competition III dataset IIIA for the subject K3b. Figure 8 demonstrates the sub-band-wise performance accuracy. We observed that our performance accuracy of the four combined subband features is better than that of the individual band features. This study advances the understanding of BCI by demonstrating the fact that robust feature selection and classification methods can significantly improve the performance of MI classification tasks. Relief-F for feature selection in conjunction with LDA for classification provides a framework that is both effective and computationally efficient. These findings support the potential for developing more reliable and accurate BCI systems, which can have broad applications in neurorehabilitation, assistive technology, and brain-machine interfaces. Future research should explore the scalability of these methods to larger and more diverse datasets and investigate adaptive or data-driven approaches for frequency band division to enhance performance further. To further address the concern about generalizability, future work will involve validating our proposed method on larger and more diverse datasets. This will help ensure the robustness and generalizability of our results across a broader range of conditions and subjects. Additionally, we plan to explore adaptive or data-driven methods for optimal frequency band division, potentially enhancing the performance of our approach.

## 7. Conclusions

In this study, we proposed a multiband decomposed feature extraction and effective feature selection-based MI tasks classification system for BCI applications. In the procedure, we extracted CSP features from four subbands to capture frequency-specific narrowband-oriented information relevant to motor imagery. We concatenated the four subband features that produce the high-dimensional feature vector and an effective feature selection method, which we then used to reduce the feature vector’s dimensionality to improve the system’s accuracy and efficiency. The existing MI classification system still faces challenges in selecting potential features to reduce the high dimensionality of the multiband-composed features. To tackle the issue of high dimensionality and enhance classification performance, we employed the Relief-F feature selection method, which effectively reduces the feature space while retaining the most relevant features. We tested the reduced feature vector with various advanced classification methods including SVM, LDA, and MLP to identify the optimal combinations for recognizing motor imagery tasks in BCI applications. The proposed model achieved higher performance accuracy than the existing systems available in the literature. This integrated approach improves classification accuracy and reduces computational complexity, making it suitable for real-time BCI applications. Our study bridges the research gap by presenting a robust and efficient framework for MI task classification using EEG signals. Future work will explore additional machine learning and deep learning methods for precise feature selection and classification to extend these approaches to multiclass MI classification challenges within the BCI paradigm. Future research also should explore further optimization of feature selection and classification techniques and their application to other large multi-class EEG datasets. This will further enhance the applicability and performance of BCI systems in real-world scenarios.

## Figures and Tables

**Table 1:** Summary of training and testing trials.

Subject	Training Trial	Testing Trial
aa	168	112
al	224	56
av	84	196
aw	56	224
ay	28	252

**Table 2:** Performance comparison in terms of MI tasks classification accuracy on BCI competition III dataset IVA of the proposed method with state-of-the-art works. The highest accuracy is marked in boldface.

Articles	Techniques	Subjects					Mean ± SD
		aa	al	av	aw	ay	
Belwafi et al. [44]	WOLA-CSP	66.07	96.07	52.14	71.43	50.00	67.29
Dai et al. [45]	TKCSP	68.10	93.88	68.47	88.40	74.93	79.17
She et al. [46]	H-ELM	63.39	98.39	64.08	85.67	85.16	79.33
Park et al. [47]	SSS-CSP	74.11	100	67.78	90.07	89.29	84.46
Jian et al. [48]	CSP-R-MF	81.43	92.41	70.00	83.57	85.00	82.48
Selim et al. [49]	AM-BA-SVM	86.61	100	66.84	90.63	80.95	85.00
Singh et al. [50]	SR-MDRM	79.46	100	73.46	89.28	88.49	86.13
Zhang et al. [51]	MKELM	83.30	98.50	71.40	91.30	93.30	87.50
Singh et al. [52]	R-MDRM	81.25	100	76.53	87.05	91.26	87.21
Ang et al. [23]	FBCSP	-	-	-	-	-	90.03 ± 0.70
Kabir et al. [2]	SRCFS + LDA	88.03	97.98	74.17	94.76	95.31	90.05
**Proposed Method**	**Relief-F + LDA**	89.29	98.57	75.36	97.51	96.43	**91.432 ± 9.69**

**Table 3:** The effectiveness of different techniques was assessed using AUROC on the BCI competition III dataset IVA, with bold text indicating the highest outcome.

Methods + Classifiers	AUROC				
	aa	al	av	aw	ay
Relief-F + LDA	**0.9346**	**0.9992**	**0.8269**	**0.9966**	0.9878
Inf-FS + LDA	0.9532	0.9914	0.8216	0.9937	0.9855
ILFS + LDA	0.9305	0.9930	0.7945	0.9925	0.9860
FSV + LDA	0.9112	0.9911	0.8049	0.9965	0.9846
SD + LDA	0.9280	0.9936	0.7610	0.9892	**0.9883**
Relief-F + SVM	0.9289	0.9946	0.8048	0.9917	0.9867
Inf-FS + SVM	0.9280	0.9889	0.7893	0.9812	0.9808
ILFS + SVM	0.9155	0.9914	0.7235	0.9856	0.9806
FSV + SVM	0.9144	0.9818	0.7872	0.9926	0.9876
SD + SVM	0.9135	0.9916	0.7596	0.9856	0.9868
Relief-F + MLP	0.8495	0.9886	0.7431	0.9910	0.9815
Inf-FS + MLP	0.8794	0.9920	0.7639	0.9884	0.9756
ILFS + MLP	0.8621	0.9906	0.7182	0.9914	0.9848
FSV + MLP	0.8618	0.9960	0.7514	0.9859	0.9760
SD + MLP	0.8568	0.9967	0.7224	0.9932	0.9635

**Table 4:** The effectiveness of different techniques was assessed using the F1 Score on the BCI competition III dataset IVA, with bold text indicating the highest outcome.

Methods + Classifiers	F1 Score				
	aa	al	av	aw	ay
Relief-F + LDA	0.8602	**0.9823**	0.6978	**0.9747**	**0.9632**
Inf-FS + LDA	**0.8929**	0.9813	**0.7671**	0.9524	0.9527
ILFS + LDA	0.8459	0.9790	0.7214	0.9677	0.9481
FSV + LDA	0.8375	0.9754	0.7387	0.9673	0.9603
SD + LDA	0.8723	0.9790	0.7004	0.9496	0.9446
Relief-F + SVM	0.8736	0.9635	0.7224	0.9680	0.9537
Inf-FS + SVM	0.8664	0.9524	0.6855	0.9507	0.9357
ILFS + SVM	0.8244	0.9712	0.6667	0.9603	0.9416
FSV + SVM	0.8410	0.9712	0.7194	0.9500	0.9531
SD + SVM	0.8511	0.9600	0.6947	0.9531	0.9493
FSV + SVM	0.8410	0.9712	0.7194	0.9500	0.9531
SD + SVM	0.8511	0.9600	0.6947	0.9531	0.9493
Relief-F + MLP	0.2191	0.0288	0.2930	0.0699	0.0976
Inf-FS + MLP	0.1857	0.0145	0.3264	0.0435	0.0712
ILFS + MLP	0.2270	0.0144	0.3200	0.0641	0.0500
FSV + MLP	0.2071	0.0073	0.3169	0.0433	0.0969
SD + MLP	0.2308	0.0144	0.3261	0.0358	0.1103

**Table 5:** The effectiveness of different techniques was assessed using computational time in seconds, on the BCI competition III dataset IVA, with bold text indicating the best outcome.

Methods + Classifiers	Computational Time				
	aa	al	av	aw	ay
Relief-F + LDA	0.2796	**0.0134**	**0.0118**	0.0113	**0.0105**
Inf-FS + LDA	0.3307	0.0154	0.0189	0.0202	0.0108
ILFS + LDA	0.3316	0.0171	0.0139	0.0132	0.0129
FSV + LDA	0.3446	0.0187	0.0131	0.0148	0.0153
SD + LDA	0.2848	0.0152	0.0140	**0.0106**	0.0108
Relief-F + SVM	0.5114	0.0154	0.0125	0.0128	0.0131
Inf-FS + SVM	0.3214	0.0179	0.0149	0.0150	0.0162
ILFS + SVM	**0.2548**	0.0144	0.0128	0.0124	0.0139
FSV + SVM	0.3770	0.0181	0.0161	0.0136	0.0145
SD + SVM	0.2774	0.0146	0.0126	0.0119	0.0131
Relief-F + MLP	2.8526	0.2682	0.1771	0.4790	0.6512
Inf-FS + MLP	1.1610	0.9800	0.2301	0.2626	0.7226
ILFS + MLP	1.3252	0.3228	0.2240	0.2461	0.8300
FSV + MLP	1.0121	0.7912	0.1814	0.2244	0.3561
SD + MLP	1.1697	0.3335	0.2207	0.2423	0.3119

**Table 6:** The effectiveness of different techniques was assessed using AUROC on the BCI competition III dataset IIIB, with bold text indicating the highest outcome.

Methods + Classifiers	AUROC	
	S4	X11
Relief-F + LDA	**0.8576**	**0.7795**
Inf-FS + LDA	0.8571	0.7721
ILFS + LDA	0.8527	0.7661
FSV + LDA	0.0.8509	0.7714
SD + LDA	0.8478	0.7672
Relief-F + SVM	0.8402	0.7575
Inf-FS + SVM	0.8436	0.7466
ILFS + SVM	0.8392	0.7642
FSV + SVM	0.8224	0.7579
SD + SVM	0.8369	0.7595
Relief-F + MLP	0.8295	0.7423
Inf-FS + MLP	0.8413	0.7209
ILFS + MLP	0.8305	0.7509
FSV + MLP	0.8438	0.7527
SD + MLP	0.8398	0.7484

**Table 7:** The effectiveness of different techniques was assessed using F1 Score on the BCI competition III dataset IIIB, with bold text indicating the highest outcome.

Methods + Classifiers	F1 Score	
	S4	X11
Relief-F + LDA	**0.7917**	**0.7165**
Inf-FS + LDA	0.7887	0.7020
ILFS + LDA	0.7684	0.6975
FSV + LDA	0.7836	0.7063
SD + LDA	0.7747	0.7054
Relief-F + SVM	0.7553	0.6693
Inf-FS+ SVM	0.7519	0.6719
ILFS + SVM	0.7500	0.6879
FSV + SVM	0.7376	0.6759
SD + SVM	0.7233	0.6693
Relief-F + MLP	0.7815	0.7423
Inf-FS + MLP	0.2621	0.3364
ILFS + MLP	0.2657	0.3315
FSV + MLP	0.2708	0.3363
SD + MLP	0.2791	0.3009

**Table 8:** The effectiveness of different techniques was assessed using computational time on the BCI competition III dataset IIIB, with bold text indicating the best outcome.

Methods + Classifiers	Computational Time	
	S4	X11
Relief-F + LDA	**0.2708**	**0.0130**
Inf-FS + LDA	0.2998	0.0143
ILFS + LDA	0.2868	0.0152
FSV + LDA	0.2865	0.0134
SD + LDA	0.2997	0.0153
Relief-F + SVM	0.4218	0.0182
Inf-FS + SVM	0.2763	0.0185
ILFS + SVM	0.2839	0.0189
FSV + SVM	0.3382	0.0147
SD + SVM	0.2989	0.0183
Relief-F + MLP	1.1838	0.2281
Inf-FS + MLP	1.2111	0.3208
ILFS + MLP	0.8946	0.1976
FSV + MLP	1.1406	0.2466
SD + MLP	0.9072	0.1999

## Data Availability

The proposed model is evaluated with three benchmark datasets, namely BCI Competition III dataset IIIA, BCI Competition III dataset IIIB, and BCI Competition III dataset IVA, are publicly available for research purposes. The datasets can be accessed at the links here: [BCI Competition III Dataset IIIA], [BCI Competition III Dataset IIIB], [BCI Competition III Dataset IVA].

## Abbreviations

BCI	Brain–Computer Interface
EEG	Electroencephalography
MEG	Magnetoencephalogram
fNIRS	Functional near-infrared spectroscopy (fNIRS)
fMRI	Functional Magnetic Response Imaging
MI	Motor Imagery
SSVEP	Steady-State Visual-Evoked Potential
SVM	support vector machines
MLP	Multi-layer Perceptron
LDA	Linear Discriminant Analysis
AAR	Adaptive Autoregressive
CSP	Common Spatial Pattern
NCA	Neighborhood Component Analysis
PCA	Principal Component Analysis
CSP	Common Spatial Pattern
Inf-FS	Infinite Feature Selection
ILFS	Infinite Latent Feature Selection
FSV	Feature Selective Validation
SD	Statistical Dependency
DNN	Deep Neural Network

## References

[1] Kumar S., Sharma M. (2012). BCI: Next Generation for HCI. Int. J. Adv. Res. Comput. Sci. Softw. Eng..

[2] Kabir M.H., Mahmood S., Al Shiam A., Musa Miah A.S., Shin J., Molla M.K.I. (2023). Investigating Feature Selection Techniques to Enhance the Performance of EEG-Based Motor Imagery Tasks Classification. Mathematics.

[3] Brooke Becher M.U. (2023). Builtin. https://builtin.com/hardware/brain-computer-interface-bci.

[4] Kayikcioglu T., Aydemir O. (2010). A polynomial fitting and k-NN based approach for improving classification of motor imagery BCI data. Pattern Recognit. Lett..

[5] Vaughan T.M., Heetderks W.J., Trejo L.J., Rymer W.Z., Weinrich M., Moore M.M., Kübler A., Dobkin B.H., Birbaumer N., Donchin E. (2003). Brain-computer interface technology: A review of the Second International Meeting. IEEE Trans. Neural Syst. Rehabil. Eng. Publ. IEEE Eng. Med. Biol. Soc..

[6] Thomas K.P., Guan C., Lau C.T., Vinod A.P., Ang K.K. (2009). A new discriminative common spatial pattern method for motor imagery brain–computer interfaces. IEEE Trans. Biomed. Eng..

[7] Miah A.S.M., Shin J., Hasan M.A.M., Molla M.K.I., Okuyama Y., Tomioka Y. Movie oriented positive negative emotion classification from EEG signal using wavelet transformation and machine learning approaches. Proceedings of the 2022 IEEE 15th International Symposium on Embedded Multicore/Many-core Systems-on-Chip (MCSoC).

[8] Miah A.S.M., Islam M.R., Molla M.K.I. EEG classification for MI-BCI using CSP with averaging covariance matrices: An experimental study. Proceedings of the 2019 International Conference on Computer, Communication, Chemical, Materials and Electronic Engineering (IC4ME2).

[9] Miah A.S.M., Shin J., Islam M.M., Molla M.K.I. Natural Human Emotion Recognition Based on Various Mixed Reality (MR) Games and Electroencephalography (EEG) Signals. Proceedings of the 2022 IEEE 5th Eurasian Conference on Educational Innovation (ECEI).

[10] Miah A.S.M., Mouly M.A., Debnath C., Shin J., Sadakatul Bari S. (2021). Event-Related Potential Classification based on EEG data using xDWAN with MDM and KNN. Proceedings of the International Conference on Computing Science, Communication and Security.

[11] Miah A.S.M., Ahmed S.R.A., Ahmed M.R., Bayat O., Duru A.D., Molla M.K.I. Motor-Imagery BCI task classification using riemannian geometry and averaging with mean absolute deviation. Proceedings of the 2019 Scientific Meeting on Electrical-Electronics and Biomedical Engineering and Computer Science (EBBT).

[12] Lal U. (2023). Neuroscience Meets Data Science: Exploring Common Spatial Pattern (CSP) and Its Applications in Healthcare Analytics. https://medium.com/geekculture/common-spatial-pattern-and-its-applications-in-the-healthcare-industry-faa4311dab79#:~:text=CSP%20has%20been%20widely%20used,motor%20intentions%20from%20EEG%20signals.

[13] Arvaneh M., Guan C., Ang K.K., Quek C. (2013). Optimizing spatial filters by minimizing within-class dissimilarities in electroencephalogram-based brain–computer interface. IEEE Trans. Neural Netw. Learn. Syst..

[14] Aggarwal S., Chugh N. (2019). Signal processing techniques for motor imagery brain computer interface: A review. Array.

[15] Singh A., Hussain A.A., Lal S., Guesgen H.W. (2021). A comprehensive review on critical issues and possible solutions of motor imagery based electroencephalography brain-computer interface. Sensors.

[16] Blankertz B., Dornhege G., Krauledat M., Müller K.R., Curio G. (2007). The non-invasive Berlin brain–computer interface: Fast acquisition of effective performance in untrained subjects. NeuroImage.

[17] Pfurtscheller G., Neuper C., Schlogl A., Lugger K. (1998). Separability of EEG signals recorded during right and left motor imagery using adaptive autoregressive parameters. IEEE Trans. Rehabil. Eng..

[18] Ghanbar K.D., Rezaii T.Y., Farzamnia A., Saad I. (2021). Correlation-based common spatial pattern (CCSP): A novel extension of CSP for classification of motor imagery signal. PLoS ONE.

[19] Joy M.M.H., Hasan M., Miah A.S.M., Ahmed A., Tohfa S.A., Bhuaiyan M.F.I., Zannat A., Rashid M.M. (2020). Multiclass mi-task classification using logistic regression and filter bank common spatial patterns. Proceedings of the International Conference on Computing Science, Communication and Security.

[20] Zhang S., Sun L., Mao X., Hu C., Liu P. (2021). Review on EEG-based authentication technology. Comput. Intell. Neurosci..

[21] Ramos A.C., Hernández R.G., Vellasco M. Feature selection methods applied to motor imagery task classification. Proceedings of the 2016 IEEE Latin American Conference on Computational Intelligence (LA-CCI).

[22] Miah A.S.M., Rahim M.A., Shin J. (2020). Motor-imagery classification using riemannian geometry with median absolute deviation. Electronics.

[23] Ang K.K., Chin Z.Y., Zhang H., Guan C. Filter bank common spatial pattern (FBCSP) in brain-computer interface. Proceedings of the 2008 IEEE International Joint Conference on Neural Networks (IEEE World Congress on Computational Intelligence).

[24] Molla M.K.I., Al Shiam A., Islam M.R., Tanaka T. (2020). Discriminative feature selection-based motor imagery classification using EEG signal. IEEE Access.

[25] Venkatachalam K., Devipriya A., Maniraj J., Sivaram M., Ambikapathy A., Iraj S.A. (2020). A Novel Method of motor imagery classification using eeg signal. Artif. Intell. Med..

[26] Tiwari A., Chaturvedi A. (2023). Automatic EEG channel selection for multiclass brain-computer interface classification using multiobjective improved firefly algorithm. Multimed. Tools Appl..

[27] Blankertz B., Muller K.R., Krusienski D.J., Schalk G., Wolpaw J.R., Schlogl A., Pfurtscheller G., Millan J.R., Schroder M., Birbaumer N. (2006). The BCI competition III: Validating alternative approaches to actual BCI problems. IEEE Trans. Neural Syst. Rehabil. Eng..

[28] (2004). Dataset IIIb: Non-Stationary 2-Class BCI Data. https://www.bbci.de/competition/iii/#data_set_iiib.

[29] Miah A.S.M., Islam M.R., Molla M.K.I. Motor imagery classification using subband tangent space mapping. Proceedings of the 2017 20th International Conference of Computer and Information Technology (ICCIT).

[30] Yu H., Ba S., Guo Y., Guo L., Xu G. (2022). Effects of motor imagery tasks on brain functional networks based on EEG Mu/Beta rhythm. Brain Sci..

[31] Wang F., Liu H., Zhao L., Su L., Zhou J., Gong A., Fu Y. (2022). Improved brain–computer interface signal recognition algorithm based on few-channel motor imagery. Front. Hum. Neurosci..

[32] Velasco I., Sipols A., De Blas C.S., Pastor L., Bayona S. (2023). Motor imagery EEG signal classification with a multivariate time series approach. BioMed. Eng. OnLine.

[33] Chaudhary S., Taran S., Bajaj V., Siuly S. (2020). A flexible analytic wavelet transform based approach for motor-imagery tasks classification in BCI applications. Comput. Methods Programs Biomed..

[34] Relief (Feature Selection). https://en.wikipedia.org/wiki/Relief_%28feature_selection%29#cite_note-1.

[35] Roffo G., Melzi S., Cristani M. Infinite feature selection. Proceedings of the IEEE International Conference on Computer Vision.

[36] Roffo G., Melzi S., Castellani U., Vinciarelli A. Infinite latent feature selection: A probabilistic latent graph-based ranking approach. Proceedings of the IEEE International Conference on Computer Vision.

[37] Bongiorno J., Mariscotti A. (2022). Uncertainty and Sensitivity of the Feature Selective Validation (FSV) Method. Electronics.

[38] Degirmenci M., Yuce Y.K., Perc M., Isler Y. (2023). Statistically significant features improve binary and multiple Motor Imagery task predictions from EEGs. Front. Hum. Neurosci..

[39] ML|Linear Discriminant Analysis. https://www.geeksforgeeks.org/ml-linear-discriminant-analysis//.

[40] Izenman A. (2013). Linear Discriminant Analysis in Modern Multivariate Statistical Techniques.

[41] Hearst M.A., Dumais S.T., Osuna E., Platt J., Scholkopf B. (1998). Support vector machines. IEEE Intell. Syst. Their Appl..

[42] Bento C. (2021). Multilayer Perceptron Explained with a Real-Life Example and Python Code: Sentiment Analysis. https://towardsdatascience.com/multilayer-perceptron-explained-with-a-real-life-example-and-python-code-sentiment-analysis-cb408ee93141#:~:text=Multilayer%20Perceptron%20is%20a%20Neural,linear%20and%20non%2Dlinear%20data&text=This%20is%20the%20first%20article,dating%20back%20to%20the%201940’s.

[43] Meng M., Dong Z., Gao Y., She Q. (2023). Optimal channel and frequency band-based feature selection for motor imagery electroencephalogram classification. Int. J. Imaging Syst. Technol..

[44] Belwafi K., Romain O., Gannouni S., Ghaffari F., Djemal R., Ouni B. (2018). An embedded implementation based on adaptive filter bank for brain–computer interface systems. J. Neurosci. Methods.

[45] Dai M., Zheng D., Liu S., Zhang P. (2018). Transfer kernel common spatial patterns for motor imagery brain-computer interface classification. Comput. Math. Methods Med..

[46] She Q., Chen K., Ma Y., Nguyen T., Zhang Y. (2018). Sparse representation-based extreme learning machine for motor imagery EEG classification. Comput. Intell. Neurosci..

[47] Park Y., Chung W. BCI classification using locally generated CSP features. Proceedings of the 2018 6th International Conference on Brain-Computer Interface (BCI).

[48] Feng J.K., Jin J., Daly I., Zhou J., Niu Y., Wang X., Cichocki A. (2019). An optimized channel selection method based on multifrequency CSP-rank for motor imagery-based BCI system. Comput. Intell. Neurosci..

[49] Selim S., Tantawi M.M., Shedeed H.A., Badr A. (2018). A csp\am-ba-svm approach for motor imagery bci system. IEEE Access.

[50] Singh A., Lal S., Guesgen H.W. (2019). Reduce calibration time in motor imagery using spatially regularized symmetric positives-definite matrices based classification. Sensors.

[51] Zhang Y., Wang Y., Zhou G., Jin J., Wang B., Wang X., Cichocki A. (2018). Multi-kernel extreme learning machine for EEG classification in brain-computer interfaces. Expert Syst. Appl..

[52] Singh A., Lal S., Guesgen H.W. (2019). Small sample motor imagery classification using regularized Riemannian features. IEEE Access.

[53] Wang M., Zhou H., Li X., Chen S., Gao D., Zhang Y. (2023). Motor imagery classification method based on relative wavelet packet entropy brain network and improved lasso. Front. Neurosci..

